# Relapsing polychondritis: clinical presentations, disease activity and outcomes

**DOI:** 10.1186/s13023-014-0198-1

**Published:** 2014-12-20

**Authors:** Aman Sharma, Arjun Dutt Law, Pradeep Bambery, Vinay Sagar, Ajay Wanchu, Varun Dhir, Rajesh Vijayvergiya, Kusum Sharma, Ashok Gupta, Naresh K Panda, Surjit Singh

**Affiliations:** Department of Internal Medicine, Postgraduate Institute of medical education and research, Chandigarh, India; Department of Cardiology, Postgraduate Institute of medical education and research, Chandigarh, India; Department of Medical Microbiology, Postgraduate Institute of medical education and research, Chandigarh, India; Department of Otorhinolaryngology, Postgraduate Institute of medical education and research, Chandigarh, India; University of Queensland, Bundaberg Hospital, Bundaberg, Australia; Division of Arthritis and Rheumatic Diseases, Oregon Health & Science University, Portland, Oregon USA

**Keywords:** Relapsing polychondritis, RPADAI, Auricular chondritis, MAGIC syndrome, Survival

## Abstract

**Background:**

Relapsing polychondritis is a rare disease characterised by inflammation of cartilaginous and proteoglycan rich structures. As there are only a few published single centre case series from all across the world, we describe our experience with 26 patients at a tertiary centre in north India.

**Methods:**

A retrospective study with all patients meeting Damiani and Levine’s modification of McAdam’s diagnostic criteria. Clinical details, investigations, disease activity assessment [(Relapsing Polychondritis Disease Activity Index (RPDAI)], treatment and outcomes were recorded.

**Results:**

Ten men and sixteen women (median age 45 years) met the diagnostic criteria. Auricular chondritis (96%), arthritis (54%), hearing impairment (42%), ocular (42%), dermal (26%), cardiovascular (11%) and laryngotracheal involvement (11%) characterized the clinical presentations. The median RPDAI was 31 (range 9-66). Two patients died during observation. Overall survival was 92.3% (median survival 13.5 years).

**Conclusions:**

Apart from reduced laryngotracheal involvement, RP in India was clinically similar to recorded patterns elsewhere.

## Background

Relapsing Polychondritis (RP,Orpha 728) is usually a recurring, autoimmune, inflammatory disorder predominantly affecting cartilage and other proteoglycan rich structures, with clinically significant ear, nose, articular and tracheobronchial tree involvement. Significantly, the eyes, heart and blood vessels may also be involved to a variable degree. Although rare, it may be associated with significant morbidity and mortality especially if associated with respiratory tract or cardiovascular involvement. Its rarity however, is offset by typical clinical features that enable a rapid diagnosis. Fortunately, it usually responds rapidly and dramatically to immunosuppressive therapy. Case series from western nations defined its clinical spectrum [1-3]. Smaller series from south-east Asia and India have, however, depicted clinical presentations at variance from those in Caucasian patients [4-6] (see Table 1). To document these differences, we present the largest single centre experience from the Indian subcontinent.

**Table 1:** **Comparison of present study patient characteristics with previously published data**

**Parameter**	**McAdam et al.** **[** 1 **]**	**Michet et al.** **[** 2 **]**	**Trentham & Le** **[** 3 **]**	**Kong et al.** **[** 4 **]**	**Ananthakrishna et al.** **[** 6 **]**	**Mathew et al.** **[** 7 **]**	**Present study**
Year of Publication	1976	1986	1998	2003	2009	2012	2014
No. of patients	159	112	66	12	7	30	26
Mean age at diagnosis	44	51	46	34	40	43	45
Female: Male	76:83	55:57	49:17	3:1	2.5:1	23:20	1.6:1
Auricular Chondritis (%)	89	85	95	83	57	88	96.1
Saddle Nose (%)	NR	29	20	17	NR	NR	11.5
Arthritis (%)	81	52	85	75	43	60	53.8
Hearing impairment (%)	46	30	42	17	NR	37	46.1
Ocular involvement (%)	65	51	57	67	43	57	42.3
Laryngeal/tracheal involvement (%)	56	48	67	50	43	37	11.5
Cardiovascular involvement (%)	9	6	8	8	14.3^*^	NR	11.5
Skin involvement (%)	17	28	38	0	14.3^*^	NR	26.9
Tracheostomy (%)	6	NR	5	42	NR	NR	1^*^
Death (%)	6	NR	3	0	0	NR	8

*Single patient. Percentage may not be significant.

## Methods

The study was performed at the Postgraduate Institute of Medical Education and Research, a tertiary care centre in northern India. The data of all patients diagnosed with relapsing polychondritis registered with the Rheumatology department from 1997 – 2014 was collected and analysed. Twenty six patients who met the diagnostic criteria defined by McAdam and modified by Damiani and Levine were included in this analysis [1,8]. All patients underwent an extensive diagnostic evaluation including a medical history, detailed physical examination including an otolaryngological evaluation, assessment of baseline haematological and metabolic parameters, imaging evaluation of affected sites including transthoracic echocardiography and, where indicated, angiography. Laboratory evaluation included antinuclear antibody, anti neutrophil cytoplasmic antibodies and rheumatoid factor. The disease severity at presentation was assessed by using the Relapsing Polychondritis Disease Activity Index (RPDAI) [9]. This scoring system takes into account the disease manifesetations in a 28 day period. The patients were followed up regularly in the outpatients department as well as during any admission. The study protocol was approved by the institute ethics committee.

## Results

Twenty six patients (10 males, 16 females) aged 14-65 years (median 45 years) were included in this analysis. The median duration of symptoms, prior to diagnosis, was 2.5 months (7 days -5 years).

### Clinical features

Aural and nasal cartilage involvement dominated the clinical presentation. Auricular chondritis was present in twenty five patients (96.1%). Recurrent episodes were noted in five (19.2%). A surgical procedure had been performed in three patients (11.5%). Objective evidence of hearing loss was noted in twelve patients (46.15%). One patient had severe bilateral sensorineural hearing loss. Nasal symptoms were reported by twenty one patients (81%) The sequel of nasal bridge collapse was reported in three of them (11.5%). Tracheobronchial involvement was seen in three patients necessitating tracheostomy in one of them.

Ocular involvement in the form of scleritis was seen in eleven patients (42.3%) whereas two patients (7.7%) had evidence of uveitis alone.

Arthritis in fourteen patients (53.8%), skin rash in seven patients (26.9%), oral and genital ulcers in four patients (15.4%) were other frequently observed manifestations.

Cardiovascular involvement in the form of aortic aneurysm or aortic annular dilatation was seen in three patients (11.5%).

Coexistent autoimmune disorders were seen in four patients (15.4%), three of whom met criteria for SLE. One patient had nephrotic range proteinuria and was diagnosed to have minimal change disease on renal biopsy. One patient had features of multicentricreticulohistiocytosis. Three patients (11.5%) developed vasculitis- ANCA associated vasculitis in one, cutaneous vasculitis in one patient with SLE and retinal vasculitis in one patient. One of these patients with cutaneous vasculitis had SLE.

Fourteen patients (53.8%) had a low haemoglobin below 120 g/L. Leucocyte counts, platelet count and metabolic parameters were normal in all patients. The median baseline RPDAI score was 31 (range 9 – 66).

Patients were followed up regularly in the clinic as well as during any admission. The clinical course and therapeutic variables of three patients is included below to depict our experience.

### Case 1

A 47 year old female presented with a 6 month history of fever, pain and swelling in both ears and hoarseness accompanied by anorexia and a 10 Kg weight loss. She also had pain and swelling of the small joints of her hands, ankles and shoulders for the last 3 months with early morning stiffness lasting over 2 hours. Physical examination revealed acute and chronic inflammatory changes in both ears with saddle nose deformity of the nasal bridge. Computerised tomography of the neck showed evidence of thyroid cartilage destruction. RPDAI at the baseline was 38. She was managed with oral prednisolone (1 mg/Kg/day), along with azathioprine (2 mg/Kg/day) with prompt resolution of all symptoms. She was weaned off steroids and continued on azathioprine for 5 years. Azathioprine withdrawalresulted in relapse with bilateral auricular chondritis necessitating reintroduction of both steroids and azathioprine. Presently, she is only on azathioprine 150 mg/day, and is doing well after a total follow up of eight years and five months.

### Case 2

A 60 year old male was admitted with 1 month of fever, redness of both eyes, pain in the nose and throat and multiple small and large joint pains severe enough to limit mobility. While no auricular involvement was noted at admission, it developed within 24 hours. Examination revealed bilateral episcleritis, nasal and auricular inflammation. Aortic regurgitation, clinically identified, was confirmed by transthoracic echocardiography and CT angiography. Aortic root diameter was 40 mm. Radiological evidence of thyroid cartilage involvement was present as was sensorineural hearing loss. RPDAI at baseline was 54. The patient received three pulses of one gram methyl prednisolone, along with a pulse of cyclophosphamide. This was followed by oral prednisolone at a dose of 1 mg/kg/day, which was gradually tapered over ten months. He received six monthly pulses of intravenous cyclophosphamide followed by azathioprine. As significant transaminitis developed, azathioprinewas replaced by mycophenolate mofetil. After four years on it, he developed severe arthritis, so it was replaced by methotrexate. After 8 years, he is presently symptom free, on 15 mg/week of methotrexate and there has been no further increase in aortic root diameter.

### Case 3

A 47 year old male presented with a history of recurrent oral and genital ulcers with severe auricular and nasal chondritis. He also had anterior uveitis and severe bilateral large and small joint arthritis rendering him wheelchair bound. A diagnosis of MAGIC syndrome was made. RPDAI, at baseline, was 19. He was started on oral prednisolone at a dose of 1 mg/kg/day resulting in major symptomatic improvement. Azathioprine was added as a steroid sparing agent at a dose of 150 mg per day. At 12 months, he was able to resume work, steroids were stopped after six months and azathioprine, continued.

### Treatment

All patients were managed with immunosuppressive therapy with corticosteroids being the first line in all patients. Three patients required “pulse” cyclophosphamide for exacerbations of disease and azathioprine, methotrexate and mycophenolatemofetil were used in four (15.4%), three (11.5%) and one patient (3.8%) respectively. The three patients with concomitant SLE received hydroxychloroquinesulphate in addition to steroids and other immunosuppression.

At the time of writing this report, two patients died during follow up. One patient succumbed to complications of pneumonia whereas the other patient had a sudden cardiac death, possibly related to disease activity.

**Figure 1:**
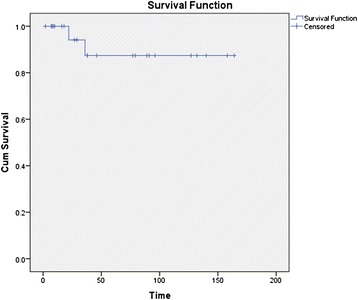
**Kaplan Meier survival curve of relapsing polychondritis (n = 26).**

The overall survival was 92.3% and the median survival was 13.5 years (Figure 1).

## Discussion

Relapsing polychondritis is associated with multiple clinical manifestations, all of which may not be identified at the time of diagnosis. The key pathogenic mechanism is the development of autoimmunity against cartilage components including types II, IX and XI collagens as well as other constituent proteins such as matrilin-1 [10]. At a cellular level, it is likely that Th1 cells play a major role, as evidenced by levels of interferon-γ, interleukin-12 and interleukin-2 correlating with disease activity [11].

There is evidence of variability of disease presentation in different ethnic populations. In a series of twelve patients of south-east Asian and ethnic Chinese origin, airway complications were more severe and cutaneous, renal and neurological complications less frequent as compared to Caucasian populations [4]. Two series have been published from the Indian subcontinent. A study of seven patients from southern India reported a reduced frequency of ear and skin involvement as compared to western data [6]. A series of ten patients from our centre had shown less frequent airway related manifestations [5].

A genetic association has been described between RP and HLA-DR4 in a study conducted in a series of 60 patients from Europe [12].

The median age of patients in this series was similar to that reported in most other studies [12]. The youngest patient in this series was 14 years old. Childhood RP has been described and its clinical presentations are similar to adult RP [13-15]. A family history of autoimmune disease is often noted in these patients although they infrequently have coexistent autoimmune diseases themselves [15].

The presence of auricular inflammation is characteristic of RP and is an important diagnostic feature. The inflammation typically spares the auricular lobe as it is devoid of cartilage and this sign can aid in its differentiation from other causes of auricular inflammation. This manifestation was noted in nearly all patients at the time of diagnosis (96.1%) in the present series and has been reported in over 80% of patients in other studies as well [12]. Multiple episodes of inflammation can result in permanently deformed ears due to loss of cartilage commonly described as “cauliflower ears”. Involvement of nasal cartilage can be seen in up to 56.5% of cases as reported in the study by Zeuner *et al*. [12]. Our series showed a higher incidence of 81% over the course of disease. Saddle nose deformity was seen in 11.5% patients. The incidence of arthritis was similar to previously published data.

Ocular involvement was noted in 42.3% patients in this series. This number is higher than the incidence reported in a series of southern Indian patients but similar to that reported in south-east Asian and Caucasian populations. Severe inflammatory changes affecting ocular tissues can lead to permanent damage and loss of vision. Any part of the eye can be affected. Scleritis can be particularly challenging to manage and may lead to globe perforation [16]. Keratitis may also occur, leading to corneal perforation [17].

Involvement of the tracheobronchial tree has been reported to be present in approximately half of all patients in western series [1,18]. The incidence of laryngeal, tracheal or bronchial involvement was 12% in our patients contrasting with the higher rates reported in other populations.

The coexistence of features of Behcet’s disease and RP has been designated as MAGIC syndrome (Mouth And Genital ulcers with Inflamed Cartilage) [19]. Three patients in our series met the criteria for this diagnosis.

Renal disease has been described in RP. In a series of 129 cases from the Mayo Clinic, renal disease was identified in 23% of cases based on the presence of hematuria, proteinuria or histopathological findings [20]. The lesions commonly identified on biopsy include mild mesangial proliferation followed by focal and segmental glomerulonephritis with crescent formation. Proteinuria in the nephrotic range was identified in 14% cases in the series by Michet *et al*. [2]. One patient had nephrotic range proteinuria in our series and was found to have features of minimal change disease on renal biopsy.

Parenchymal lung involvement was seen in one patient in our series. This exceedingly rare manifestation has not been described previously in literature and showed a dramatic response to pulse methylprednisolone [21].

An increased risk of myelodysplastic syndrome (MDS) may exist in patients with RP [22,23]. In a single centre series of 200 patients from France, MDS was seen in 11% [24]. Sweet’s syndrome has also been reported [25]. No patient, in this study, has developed a major haematological complication to date.

**Figure 2:**
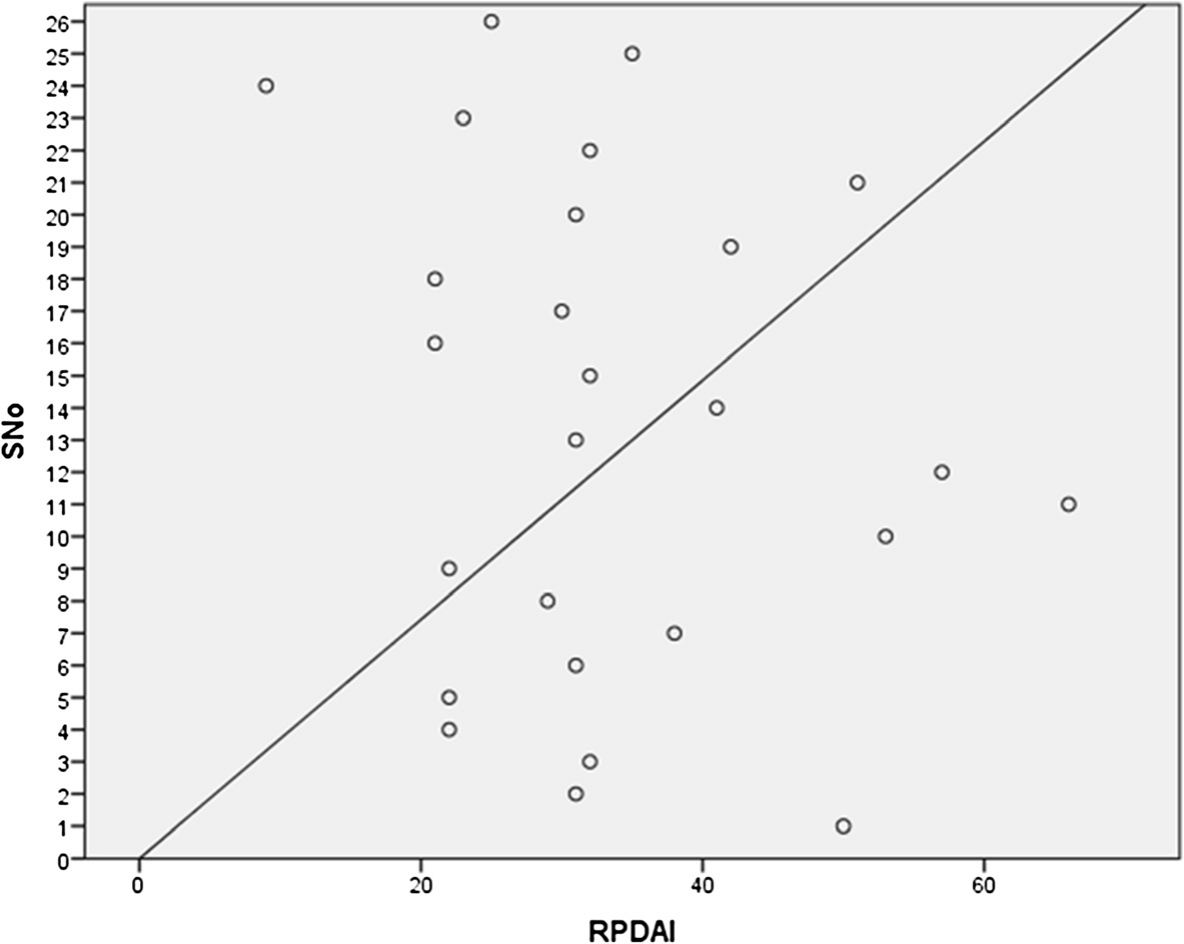
**Scatter plot of patients showing RPDAI scores.**

Relapsing polychondritis often has a fluctuating course and disease progression can be difficult to predict. A scoring system has been devised in order to provide an objective means of assessment of disease severity [9]. Using this scoring system, our cohort of patients demonstrated a median baseline RPDAI score of 31 (range 9 – 66) (Figure 2).

The emerging role of nuclear medicine based imaging has been a subject of interest and has a potential role in the diagnosis and evaluation of disease activity in RP due to its ability to identify subclinical lesions prior to the onset of frank symptoms [26].

The mainstay of therapy revolves around control of inflammation and suppression of autoimmunity. The choice of therapy is dependent on the severity of symptoms and the extent of disease. Localised disease with mild symptoms affecting the ear, nose or joints may be treated with non steroidal anti-inflammatory drugs (NSAIDS). For more severe symptoms, organ threatening or multiple system disease corticosteroids such as oral prednisolone (0.5 – 1.0 mg/kg/day) or “pulse” methylprednisolone (1000 mg/day) may be appropriate. Dapsone alone or in conjunction with steroids may be considered in milder forms of disease [8]. Other agents such as cyclophosphamide, azathioprine, methotrexate, cyclosporine, colchicine and chlorambucil may also be considered as second line options in case of organ or life threatening disease or as steroid sparing agents. In our series, pulse methylprednisolone was used in a patient with severe cardiovascular complications (aortic root dilatation) leading to excellent response [27]. Pulse cyclophosphamide was used in three patients with severe and recalcitrant disease.

Emerging treatment options include the use of immunomodulatory agents such as infliximab, etanercept, adalimumab and abatacept [28-31]. Tocilizumab, has shown utility in patients with disease unresponsive to TNF antagonists [32]. Allogeneic and autologous hematopoietic stem cell transplantation remains a controversial subject, but may be of potential utility in refractory cases [33].

The life expectancy of patients with RP may be reduced due to its progressive nature, however due to the advent of better therapeutic modalities and early diagnosis, the survival at 8 years may be as high as 94% [3].

Our study is limited by the relatively small number of patients and the referral bias inherent with a study conducted at an tertiarycentre.

## Conclusions

Relapsing polychondritis is a progressive and potentially lethal autoimmune disease affecting multiple organs and tissues. Early recognition of the signs of this disease and prompt initiation of anti-inflammatory and immunosuppressive therapy can ameliorate symptoms and prevent end organ complications, particularly those affecting the respiratory tract and the heart.

Emerging diagnostic modalities such as FDG-PET scanning and newer immunosuppression strategies provide hope for improved outcomes. The RPDAI may provide an accurate and objective estimation of disease activity.
